# Fetal liver Mll-AF4+ hematopoietic stem and progenitor cells respond directly to poly(I:C), but not to a single maternal immune activation

**DOI:** 10.1016/j.exphem.2019.07.004

**Published:** 2019-08

**Authors:** Camille Malouf, Katrin Ottersbach

**Affiliations:** MRC Centre for Regenerative Medicine, University of Edinburgh, Edinburgh EH16 4UU, United Kingdom

## Abstract

T(4;11) MLL-AF4 acute leukemia is one of the most aggressive malignancies in infant and pediatric populations. Epidemiological and functional studies have highlighted the influence of an overstimulation of the immune system on leukemia development. This study aimed at assessing if the cell-of-origin of t(4;11) MLL-AF4 acute leukemia is sensitive to a viral or bacterial mimic and if maternal immune activation can lead to a full-blown leukemia. To answer this, we used the Mll-AF4 pre-leukemia mouse model that initiates the expression of Mll-AF4 in the first definitive hematopoietic cells formed during embryonic development. We observed an increase in proliferation upon hematopoietic differentiation of fetal liver Mll-AF4+ Lineage–Sca1+ckit+ (LSK) cells exposed to the immune stimulants, poly(I:C) or LPS/lipopolysaccharide. This was accompanied by increased expression of a subset of MLL-AF4 signature genes and members of the Toll-like receptor signaling pathways in fetal liver Mll-AF4+ LSK exposed to poly(I:C), suggesting that the cell-of-origin responds to inflammatory stimuli. Maternal immune activation using a single dose of poly(I:C) did not lead to the development of leukemia in Mll-AF4+ and control offspring. Instead, aging MLL-AF4+ mice showed an increased proportion of T-lymphoid cells in the spleen, lost their B-lymphoid bias, and had decreased frequencies of hematopoietic stem and multipotent progenitor cells. Overall, this study suggests that the fetal liver Mll-AF4+ LSK cells are sensitive to direct exposure to inflammatory stimuli, especially poly(I:C); however, maternal immune activation induced by a single exposure to poly(I:C) is not sufficient to initiate MLL-AF4 leukemogenesis.

## Highlights

• Poly(I:C) and LPS promote the proliferation of fetal Mll-AF4+ B-lymphoid cells.
• Poly(I:C) is a positive regulator of the Mll-AF4 expression signature.
• A single maternal immune activation does not induce Mll-AF4+ leukemia.
• The Mll-AF4-induced B-lymphoid bias disappears with aging.
• Mll-AF4 does not support the long-term maintenance of early progenitors.

T(4;11) MLL-AF4 acute lymphoblastic leukemia is an aggressive subtype of infant and pediatric leukemia that originates in utero, with monozygotic twin studies having reported a 100% penetrance [1]. We are starting to gain more insight into how the disease develops through the use of pre-leukemia and leukemia mouse models 2, 3, 4, 5, 6, 7, 8. Using a pre-leukemia mouse model, in which expression of Mll-AF4 initiates in all definitive hematopoietic cells formed during embryonic development (Mll-AF4 invertor mouse crossed with VEC-Cre), we previously identified the fetal liver as the starting point of MLL-AF4-driven leukemogenesis 4, 7. At this stage, Mll-AF4 expression increases the engraftment and self-renewal potential of hematopoietic stem and immature progenitor cells (Lineage–Sca1+ckit+ [LSK] cells), but also induces a high B-lymphoid clonogenic bias.

The etiology of MLL-AF4 infant and pediatric leukemia is largely unknown. One theory in the pediatric leukemia field is that leukemogenesis requires additional stress signals, such as an overstimulation of the inflammatory response 9, 10, 11. Although there is strong evidence supporting the role of infections as triggers of leukemia in older children, it is currently unknown whether an abnormal stimulation of the immune system during gestation also triggers leukemia in infants. We therefore decided to investigate if fetal liver Mll-AF4+ LSK cells from the pre-leukemia mouse model were sensitive to viral or bacterial mimics through use of the double-stranded RNA viral analog polyinosinic:polycytidylic acid (poly(I:C)) or the bacterial endotoxin lipopolysaccharide (LPS). These molecules bind the Toll-like receptors Tlr3 and Tlr4, respectively, which are crucial to adaptive immunity (reviewed in [12]). They can also increase the proliferation of adult hematopoietic stem and progenitor cells 13, 14, 15. We assessed how both mimics influence myeloid and B-lymphoid clonogenic potential, differentiation, and proliferation, but also the expression of MLL-AF4 signature genes. Although in vitro stimulation of fetal liver Mll-AF4+ LSK cells with poly(I:C) or LPS had no effect on myeloid or B-lymphoid hematopoietic clonogenic potential, poly(I:C) was able to increase proliferation in myeloid and B-lymphoid conditions, whereas LPS increased proliferation in B-lymphoid conditions only. In addition, exposure to poly(I:C), but not LPS, upregulated the expression of MLL-AF4 signature genes (*Mll-AF4, Mll, Af4, Cdk6, Hmga2, Hoxa9, Il7r, Lmo2,* and *Mcl1*) and members of the Toll-like signaling pathways (*Tlr9, Mda5,* and *Rig1*) in fetal liver Mll-AF4+ LSK cells. We also induced a maternal immune activation (MIA) with poly(I:C) to assess its effect on MLL-AF4-driven leukemogenesis in the offspring. We found that a single intraperitoneal injection of poly(I:C) at E14–E15 was not sufficient to trigger MLL-AF4 leukemogenesis. Instead, Mll-AF4+VEC-Cre+ aging mice showed an increase of CD3+ T-lymphoid cells in the spleen, maintained their progenitor B-cell pool, and had a lower proportion of hematopoietic stem cells (HSCs) and multipotent progenitors (MPPs). This study suggests that fetal liver MLL-AF4+ LSK cells are sensitive to viral and bacterial mimics that can activate the Toll-like receptor signaling pathways, but a single maternal immune activation is not enough to initiate and/or maintain MLL-AF4-driven leukemogenesis.

## Methods

### Mice

All animal work was carried out under the regulation of the UK Home Office. The Mll-AF4 line was obtained from Professor Terry Rabbitts [2], and the VEC-Cre line from Professor Nancy Speck [16]. Males and females were mated for embryo generation, with the day of the plug counted as day 0 of embryonic development. For in vivo stimulation, pregnant females received a single intraperitoneal injection of 4 or 10 mg/kg poly(I:C) (Sigma Catalog No. P0913) at E14–E15 of embryonic development.

### Sorting of E14 fetal liver LSK cells

Fetal livers were dissected and dissociated in Flow Cytometry Staining Buffer (ThermoFisher Catalog No. 00-4222-26) using a 21G × 15-mm needle attached to a syringe (BD Microlance Catalog Nos. 10472204-X and 3000185). Dissociated fetal livers were stained using the following antibody mix in Flow Cytometry Staining Buffer (ThermoFisher Catalog No. 00-4222-26): APC anti-mouse CD3ε antibody (clone I45-2C11, Biolegend Catalog No. 100312), APC anti-mouse TER-119 antibody (clone TER119, Biolegend Catalog No. 116212), APC anti-mouse F4/80 antibody (clone BM8, Biolegend Catalog No. 123116), APC anti-mouse Nk1.1 antibody (clone PK136, Biolegend Catalog No. 108709), APC anti-mouse Ly-6G/Ly-6C (Gr-1) antibody (clone RB6-8C5, Biolegend Catalog No. 108412), PE/Cy7 anti-mouse/human CD45R/B220 antibody (clone RA3-6B2, Biolegend Catalog No. 103222), PE/Cy7 anti-mouse CD19 antibody (clone 6D5, Biolegend Catalog No. 115520), CD117 (ckit) monoclonal antibody APC-eFluor 780, eBioscience (clone 2B8, ThermoFisher Catalog No. 47-1171-80), Alexa Fluor 700 anti-mouse CD45 antibody (clone 30-F11, Biolegend Catalog No. 103128), Pacific Blue anti-mouse Ly-6A/E (Sca1) antibody (Clone E13-161.7, Biolegend Catalog No. 122519). Cells were incubated for 20 min on ice, washed twice with Flow Cytometry Staining Buffer, and resuspended in diluted SYTOX Green Nucleic Acid Stain (ThermoFisher Catalog No. S7020) to exclude dead cells. Sorting was done on a BD FACSAria II (BD Biosciences).

### In vitro treatment of E14 fetal liver LSK cells

Mll-AF4+ LSK cells were plated for 48 hours in Stem-Pro34 SFM (1×) medium (ThermoFisher Scientific Catalog No. 10639011) supplemented with the following cytokines: stem cell factor (SCF, 100 ng/mL), FLT3 ligand (50 ng/mL), and thrombopoietin (TPO, 100 ng/mL) (Peprotech Catalog Nos. 250-03, 250-31L, and 315-14). Poly(I:C) or LPS was added at 100 ng/mL (Sigma, Catalog Nos. P0913 and L3023). Mock condition is sterile phosphate-buffered saline (PBS).

### Apoptosis assay

Detection of apoptotic cells was achieved through double-staining using phycoerythrin (PE)-AnnexinV (Biolegend Catalog No. 640907) and SYTOX Blue Dead Cell Stain (ThermoFisher Catalog No. S34857). Cells were stained in the Annexin V Binding Buffer according to the manufacturer's instructions (BD Biosciences Catalog No. 556454). Data were acquired on a BD LSRFortessa (BD Biosciences).

### CFU-C assays

Myeloid and lymphoid colony-forming unit cell (CFU-C) assays were carried out in MethoCult GF M3434 and MethoCult M3630, respectively (STEMCELL Technologies Catalog Nos. 03434 and 03630). For lymphoid CFU-C assays, the following additional cytokines were added: SCF (25 ng/mL) and FLT3 ligand (20 ng/mL) (Peprotech Catalog Nos. 250-03 and 250-31L). Poly(I:C) or LPS was added at 100 ng/mL. Mock condition is sterile PBS.

### Flow cytometry analysis of peripheral blood and tissues

For flow cytometry of peripheral blood and tissues, red blood cell lysis was carried out with BD Pharm Lyse lysing solution according to the manufacturer's instructions (BD Biosciences Catalog No. 555899). Cells were stained in Flow Cytometry Staining Buffer using the following mixture of antibodies for differentiated cells: APC anti-mouse CD45 antibody (clone 30-F11, Biolegend Catalog No. 103111), eFluor450-CD11b monoclonal antibody eBioscience (clone M1/70, ThermoFisher Catalog No. 48-0112-80), Alexa Fluor 700 anti-mouse Ly-6G/Ly-6C (Gr1) antibody (clone RB6-8C5, Biolegend Catalog No. 108422), PE/Cy7 anti-mouse/human CD45R/B220 antibody (clone RA3-6B2, Biolegend Catalog No. 103222), Brilliant Violet 605 anti-mouse CD19 antibody (clone 6D5, Biolegend Catalog No. 115539), antigen presenting cell (APC)-eF780 IgM mouse monoclonal antibody (clone II/41, ThermoFisher Catalog No. 47-5790-82), PE-CD3 anti-mouse antibody (clone 145-2C11, Biolegend Catalog No. 100308). For progenitor and precursor B cells, we used the following antibodies: fluorescein isothiocyanate (FITC) anti-mouse CD43 activation-associated glycoform antibody (clone 1B11, Biolegend Catalog No. 121206); Alexa Fluor 700 anti-mouse/human CD45R/B220 (clone RA3-6B2, Biolegend Catalog No. 103231); Brilliant Violet 421 anti-mouse CD45 (c-antibody, clone 30F11, Biolegend Catalog No. 103133); PE/Cy7 anti-mouse CD24 antibody (clone M1/69, Biolegend Catalog No. 101821); APC IgM mouse monoclonal antibody (clone II/41, ThermoFisher Catalog No. 17-5790-82); Brilliant Violet 605 anti-mouse CD19 antibody (clone 6D5, Biolegend Catalog No. 115539); CD117 (ckit) monoclonal antibody APC-eFluor 780, eBioscience (clone 2B8, ThermoFisher Catalog No. 47-1171-80); and PE anti-mouse CD127 (IL7Rα) antibody (clone A7R34, Biolegend Catalog No. 135010). For hematopoietic stem and progenitor cells, we used the following antibodies: APC anti-mouse CD3ε antibody (clone I45-2C11, Biolegend Catalog No. 100312); APC anti-mouse TER-119 antibody (clone TER119, Biolegend Catalog No. 116212); APC anti-mouse F4/80 antibody (clone BM8, Biolegend Catalog No. 123116); APC anti-mouse Nk1.1 antibody (clone PK136, Biolegend Catalog No. 108709); APC anti-mouse Ly-6G/Ly-6C (Gr1) antibody (clone RB6-8C5, Biolegend Catalog No. 108412); APC anti-mouse/human CD45R/B220 antibody (clone RA3-6B2, Biolegend Catalog No. 103212); APC anti-mouse CD19 antibody (clone 6D5, Biolegend Catalog No. 115512); CD117 (ckit) monoclonal antibody APC-eFluor 780, eBioscience (clone 2B8, ThermoFisher Catalog No. 47-1171-80); Alexa Fluor 700 anti-mouse CD48 antibody (clone HM48-1, Biolegend Catalog No. 103425), PE/Cy7 anti-mouse CD150 antibody (clone TC15-12F12.2, Biolegend Catalog No. 115914); Pacific Blue anti-mouse Ly-6A/E (Sca1) antibody (clone E13-161.7, Biolegend Catalog No. 122519); FITC anti-mouse CD34 antibody (clone RAM34, BD Biosciences Catalog No. 560238); PE anti-mouse CD127 (IL7Rα) antibody (clone A7R34, Biolegend Catalog No. 135010); and biotin anti-mouse CD135 (FLT3) antibody (clone A2F10, ThermoFisher Catalog No. 13-1351-85) conjugated to Qdot655 streptavidin conjugate (ThermoFisher, Cat#Q10121MP). Cells were incubated on ice for 20 min, washed twice with Flow Cytometry Staining Buffer, and resuspended in diluted SYTOX AADvanced (ThermoFisher Catalog No. S10274) to exclude dead cells. Data were acquired on a BD LSRFortessa.

### Quantitative RT-qPCR

RNA was extracted using the RNeasy Micro Kit (Qiagen Catalog No. 74004). For reverse transcription of mRNA, we used the iScript Advanced cDNA synthesis kit for real-time reverse transcription quantitative polymerase chain reaction (RT-qPCR; Bio-Rad Laboratories Catalog No. 1725037) according to the manufacturer's instructions. Primers were designed using Primer3 and tested. Primer sequences can be found in Supplementary Table E1 (online only, available at www.exphem.org). We used the Brilliant III Ultra-Fast SYBR Green qPCR Master Mix according to the manufacturer's instructions (Agilent Catalog No. 600883). Data were acquired on a QuantStudio 7 Flex Real-Time PCR System (ThermoFisher).

### Data analysis

Flow cytometry data were analyzed using FlowJo. Graphs were generated using GraphPad Prism 6 (GraphPad Software). Statistical differences were assessed using GraphPad with a nonparametric Mann–Whitney *U* test, a nonparametric Wilcoxon paired test (RT-qPCR only), or a Gehan–Breslow–Wilcoxon test (survival curve) with a bilateral *p* value, as indicated in the figure legends (*p < 0.05, **p < 0.01, ***p < 0.001).

## Results

### Poly(I:C) and LPS increase the proliferation of hematopoietic cells derived from fetal liver Mll-AF4+ hematopoietic stem and progenitor cells in vitro

**Figure 1:**
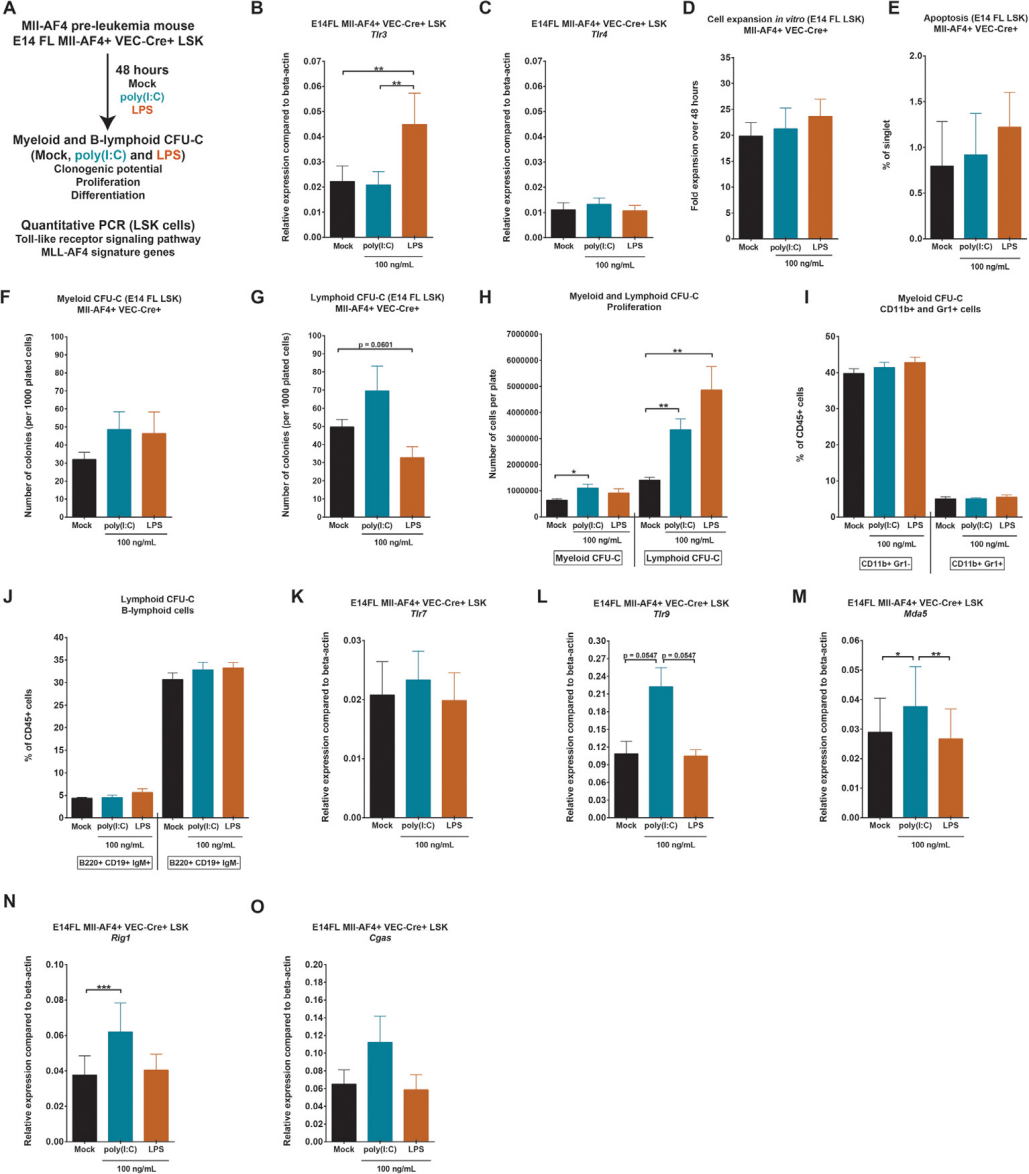
Poly(I:C) and LPS increase the proliferation of hematopoietic cells derived from fetal liver Mll-AF4+ hematopoietic stem and progenitor cells in vitro. **(A)** Experimental layout for the colony-forming assays (CFU-C) and quantitative PCR. Expression of **(B)***Tlr3* and **(C)***Tlr4* in FL Mll-AF4+ LSK cells after a 48-hour in vitro stimulation to mock/PBS, poly(I:C), or LPS. **(D)** Cell expansion of FL Mll-AF4+ LSK cells after a 48-hour in vitro stimulation to mock, poly(I:C), or LPS. **(E)** Apoptosis of FL Mll-AF4+ LSK after a 48-hour in vitro stimulation to mock, poly(I:C), or LPS. Apoptotic cells are AnnexinV+SytoxBlue–. **(F)** Myeloid or **(G)** B-lymphoid colony-forming assay of FL Mll-AF4+ LSK cells plated with mock/PBS, poly(I:C), or LPS. **(H)** Proliferation of FL Mll-AF4+ LSK cells in myeloid and B-lymphoid colony-forming assays. Differentiation of **(I)** myeloid and **(J)** B-lymphoid cells in colony-forming assays. Expression of **(K)***Tlr7*, **(L)***Tlr9*, **(M)***Mda5*, **(N)***Rig1,* and **(O)***Cgas*. Statistical differences were assessed using a nonparametric Mann–Whitney *U* test or Wilcoxon test (RT-qPCR) with a bilateral *p* value (*p < 0.05, **p < 0.01, ***p < 0.001; *n* = 3 or 4).

First, we wanted to assess the direct effect of poly(I:C) or LPS on fetal liver (FL) Mll-AF4+ hematopoietic stem and progenitor cells (LSK cells). FL Mll-AF4+ LSK cells were sorted from the MLL-AF4+ pre-leukemia mouse model according to our previous studies and plated in medium with PBS (mock condition), poly(I:C), or LPS (Figure 1A) 4, 7. After 48 hours in culture, Mll-AF4+ LSK cells were counted and plated in methylcellulose to assess the effect of poly(I:C) or LPS on myeloid and B-lymphoid clonogenic potential, proliferation, and differentiation, with continued exposure to mimics. We also collected FL Mll-AF4+ LSK exposed to poly(I:C) and LPS to measure the expression of members of the Toll-like receptor signaling pathway and MLL-AF4 signature genes.

FL Mll-AF4+ LSK expressed both *Tlr3* and *Tlr4* receptors, which are recognized by poly(I:C) and LPS, respectively (Figure 1B, C). *Tlr3* expression was significantly upregulated following LPS treatment, whereas *Tlr4* expression remained unchanged. There was no significant expansion of FL Mll-AF4+ LSK cells in vitro in the presence of poly(I:C) or LPS over the 48 hours (Figure 1D). The level of apoptosis (AnnexinV+SytoxBlue− cells) was also similar for all three conditions (Figure 1E). There was also no significant difference in the myeloid or B-lymphoid clonogenic output (Figure 1F, G). Interestingly, counting total cell numbers on CFU-C plates revealed an expansion of myeloid cells upon poly(I:C) treatment and an even more pronounced expansion of B-lymphoid cells upon either poly(I:C) or LPS treatment (Figure 1H). This was a general effect as the relative proportions of cell types were maintained (Figure 1I,J).

We assessed the expression of members of the Toll-like receptor signaling pathway in FL Mll-AF4+ LSK cells stimulated with poly(I:C) or LPS. Poly(I:C) did not affect the expression of *Tlr7*, but increased the expression of *Tlr9* (Figure 1K, L). Both Toll-like receptors are found on endosomes and are activated by the presence of viral RNA. *Mda5* and *Rig1*, two viral sensors, were also upregulated in FL Mll-AF4+ LSK cells upon poly(I:C) treatment (Figure 1M, N). *Cgas*, a double-stranded DNA sensor, maintained a similar expression upon poly(I:C) or LPS treatment (Figure 1O). Sensors of cytosolic RNA and DNA pathways such as *Tlr9, Mda5,* and *Rig1* can induce a pro-inflammatory phenotype through the activation of interferon (IFN)-stimulated genes (reviewed in [17]). These results indicate that hematopoietic cells derived from FL Mll-AF4+ LSK cells increase their proliferation in the presence of viral or bacterial stimuli, and also upregulate the expression of components of the Toll-like receptor signaling pathway; however, clonogenic potential was unaffected.

### Poly(I:C) affects the MLL-AF4 signature gene expression program in fetal liver Mll-AF4+ hematopoietic stem and progenitor cells

**Figure 2:**
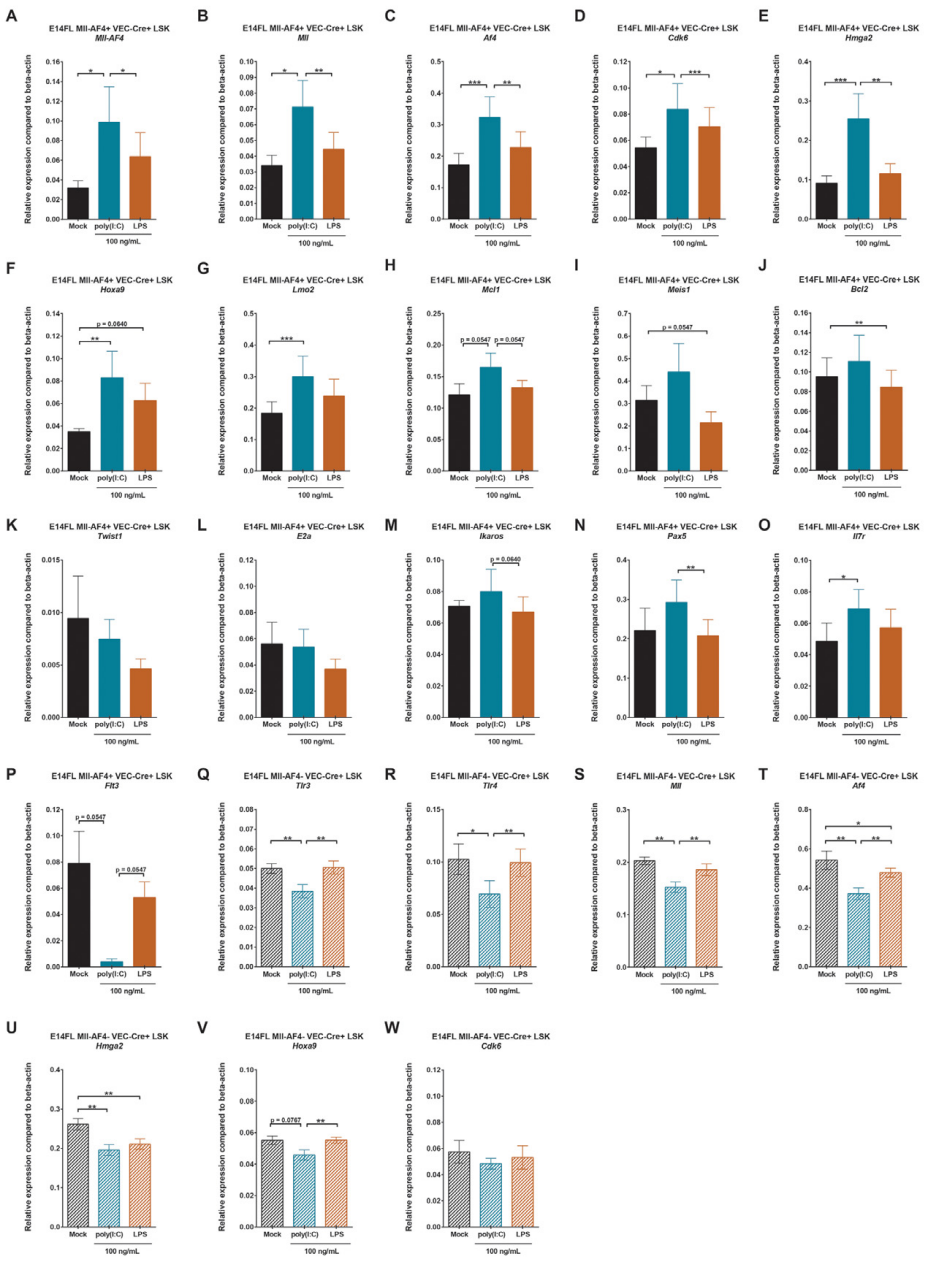
Poly(I:C) affects the MLL-AF4 signature gene expression program in fetal liver Mll-AF4+ hematopoietic stem and progenitor cells in vitro. Expression of **(A)***Mll-AF4*, **(B)***Mll*, **(C)***Af4*, **(D)***Cdk6*, **(E)***Hmga2*, **(F)***Hoxa9*, **(G)***Lmo2*, **(H)***Mcl1*, **(I)***Meis1*, **(J)***Bcl2*, **(K)***Twist1*, **(L)***E2a*, **(M)***Ikaros*, **(N)***Pax5*, **(O)***Il7r*, and **(P)***Flt3* in FL Mll-AF4+ LSK after a 48-hour in vitro stimulation with mock/PBS, poly(I:C), or LPS. Expression of **(Q)***Tlr3*, **(R)***Tlr4*, **(S)***Mll*, **(T)***Af4*, **(U)***Hmga2*, **(V)***Hoxa9*, and **(W)***Cdk6* in FL control (Mll-AF4–VEC-Cre+) LSK cells after a 48-hour in vitro stimulation with mock/PBS, poly(I:C), or LPS. Statistical differences were assessed using a nonparametric Wilcoxon test with a bilateral *p* value (*p < 0.05, **p < 0.01, *p* < 0.001; *n* = 3 or 4).

We verified the expression of a subset of genes that are involved in MLL-AF4-driven leukemogenesis in FL Mll-AF4+ LSK cells stimulated with poly(I:C) or LPS. The expression of *Mll-AF4, Mll, Af4, Cdk6, Hmga2, Hoxa9, Lmo2,* and *Mcl1* was upregulated in vitro by poly(I:C), but not LPS (Figure 2A–H). By contrast, *Meis1* and *Bcl2* were slightly downregulated upon LPS exposure, whereas poly(I:C) did not affect them (Figure 2I,J). The expression of *Twist1, E2a, Ikaros,* and *Pax5* remained similar for all three conditions (Figure 2K–N). *Il7r* expression significantly increased upon poly(I:C) exposure, which was accompanied by a downregulation of *Flt3* (Figure 2O,P). Overall*,* these results suggest that an inflammatory stimulus that mimics a viral component can directly affect the expression of MLL-AF4 signature genes in FL Mll-AF4+ LSK cells. However, as discussed earlier, these changes are not sufficient to affect the hematopoietic clonogenic or differentiation potential of FL Mll-AF4+ LSK. To determine whether this is a more general effect, independent of Mll-AF4 expression, we checked the expression of *Tlr3, Tlr4, Mll, Af4, Cdk6, Hmga2,* and *Hoxa9* in control FL LSK (Mll-AF4–VEC-Cre+) cells. Interestingly, the pattern was quite different, as an exposure to poly(I:C) led to a downregulation of *Tlr3, Tlr4, Mll, Af4, Hmga2,* and *Hoxa9* compared with mock or LPS-treated cells (Figure 2Q–V). *Cdk6* expression was stable in control FL LSK cells regardless of the condition (Figure 2W). Therefore, the Mll-AF4 expression signature can be modulated by a direct exposure to poly(I:C), but this requires the presence of the oncogenic driver.

### A poly(I:C)-induced maternal immune activation is not sufficient to drive Mll-AF4 leukemogenesis

**Figure 3:**
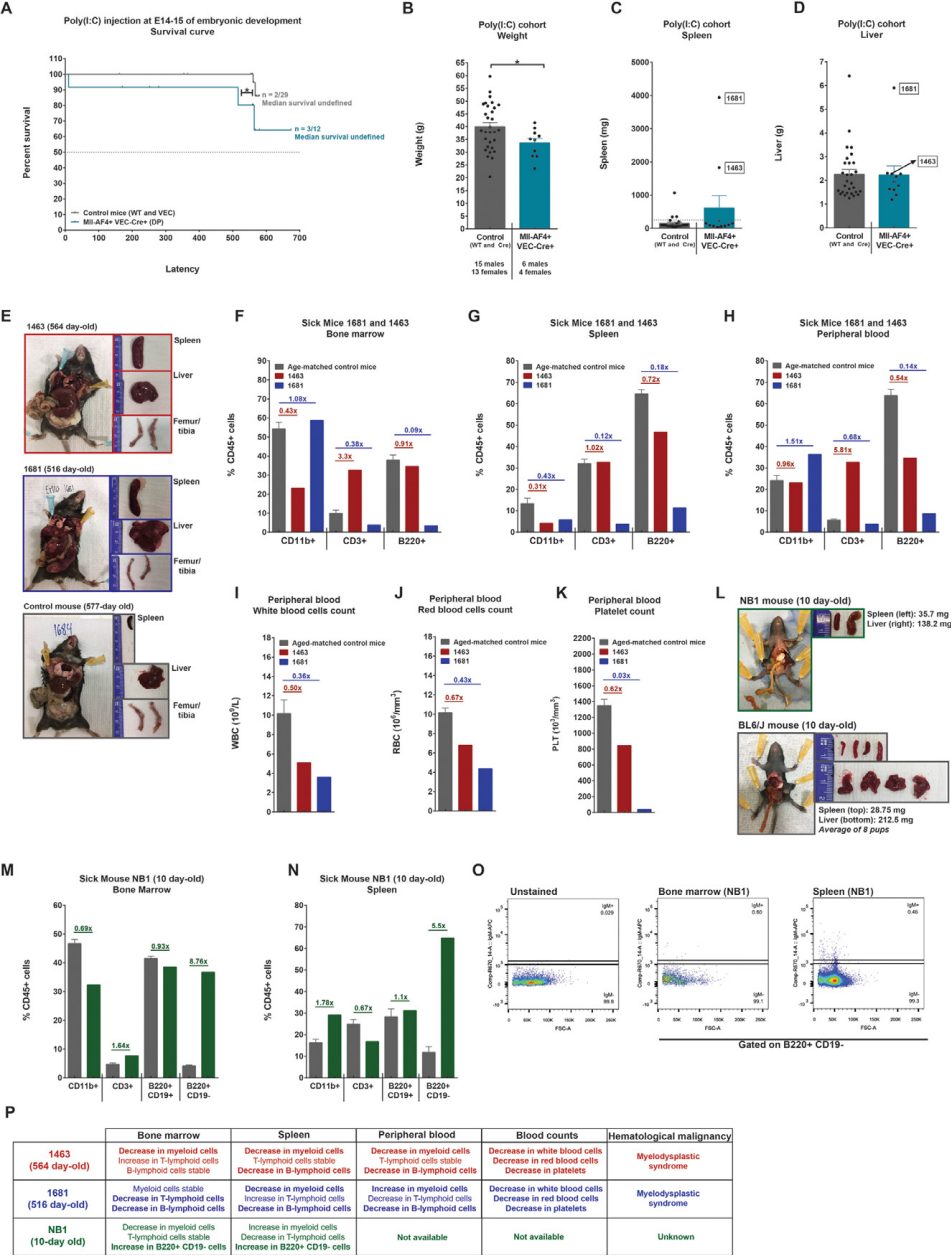
A poly(I:C)-induced maternal immune activation is not sufficient to drive Mll-AF4 leukemogenesis. **(A)** Survival curve of the progeny of pregnant dams injected with poly(I:C) at E14–E15 of embryonic development. **(B)** Body, **(C)** spleen, and **(D)** liver weights of mice at the end of study (18 months old). (**E)** Postmortem photos of sick mice 1463 and 1681 (564 and 516 days old) and control mouse 1684 (577 days old). Proportion of CD11b+ myeloid, CD3+ T-lymphoid, and B220+ B-lymphoid cells in the **(F)** bone marrow, **(G)** spleen, and **(H)** peripheral blood of sick mice 1463 and 1681. Peripheral blood counts of (**I)** white blood cells, **(J)** red blood cells, and **(K)** platelets of sick mice 1463 and 1681. **(L)** Postmortem photo of sick mouse NB1 (10 day-old) and age-matched control BL6/N mice. CD11b+ myeloid cells, CD3+ T-lymphoid cells, and B220+CD19+ and B220+CD19- B-lymphoid cells in the **(M)** bone marrow and **(N)** spleen of sick mouse NB1. **(O)** IgM expression on the B220+CD19– fraction of sick mouse NB1. **(P)** Overview of the hematopoietic features of all the sick mice (1463, 1681, and NB1). Statistical differences were assessed using a nonparametric Mann–Whitney *U* test with a bilateral *p* value or a Gehan–Breslow–Wilcoxon test (survival curve only) (*p < 0.05, **p < 0.01, ***p < 0.001).

As we found poly(I:C) to be a positive modulator of proliferation of myeloid and B-lymphoid hematopoietic cells derived from FL Mll-AF4+ LSK cells and to affect members of the MLL-AF4 signature gene expression, we decided to assess its effect on hematopoietic development in vivo. Although there is no strong evidence that poly(I:C) can penetrate the placental barrier to reach the fetal liver, the intraperitoneal injection of poly(I:C) in a pregnant dam can activate the innate immune response, leading to increased levels of cytokines in the maternal compartment (reviewed in [18]). This compromises the integrity of the maternal–fetal barrier, leading to dissemination of maternal cytokines to the embryo. Given the very short latency of infant t(4;11) MLL-AF4 acute leukemia, we wanted to assess if the maternal immune response could promote MLL-AF4-driven leukemogenesis. Pregnant dams received a single intraperitoneal injection of poly(I:C) at E14–E15 of embryonic development. This is the same developmental time point used in the in vitro analysis (Figures 1 and 2). Pups were born without complications and were monitored for a period of 18 months to assess disease development. Only 3 of 12 Mll-AF4+VEC-Cre+ mice developed a disease that was linked to a sudden death (NB1: 10 day-old) or splenomegaly (1463: 564 days old, and 1681: 516 days old) (Figure 3A). Overall, the latency in the Mll-AF4+ VEC–Cre+ cohort was significantly shorter compared with that in the Mll-AF4−VEC-Cre+/− control. Sick and end-of-study Mll-AF4+VEC-Cre+ mice had a lower weight compared with control mice (Figure 3B), but no overall significant difference in spleen or liver weight (Figure 3C,D). Mice 1463 and 1681 presented with splenomegaly, which was absent in the control mice, and a red bone marrow (Figure 3E). We analyzed the hematopoietic compartment in the bone marrow, spleen, and peripheral blood for mice 1463 and 1681 and compared the results with those of age-matched control mice. Mouse 1463 showed a decrease of CD11b+ myeloid cells in the bone marrow and spleen and a decrease of B220+ B-lymphoid cells in the spleen and peripheral blood (Figure 3F–H). Similarly, mouse 1681 showed a decrease in CD11b+ myeloid cells in the spleen and a decrease in CD3+ T-lymphoid and B220+ B-lymphoid cells in all three compartments (Figure 3F–H). In the peripheral blood, both sick mice showed a drop in white blood cell, red blood cell, and platelet counts compared with age-matched control mice (Figure 3I–K). Together, this evidence suggests that mice 1463 and 1681 developed not a leukemia, but a hematological malignancy that is similar to a myelodysplastic syndrome (Figure 3P).

NB1 mouse had a slightly enlarged spleen (35.7 mg) compared with age-matched control mice (28.75 mg) and a pale liver (Figure 3L). In the bone marrow, there was a decrease in CD11b+ myeloid cells while the numbers of CD3+ T-lymphoid cells and B220+ CD19+ B-lymphoid cells were normal (Figure 3M). Most notable was the expansion of B220+CD19– cells, which are almost absent in the control mice (Figure 3M). These B220+CD19– cells were also detected in the spleen alongside an increase in CD11b+ myeloid cells (Figure 3N). The B220+CD19– cells were also IgM– (Figure 3O). This cell population has been observed in Pax5 haploinsufficient mice that developed a pre-B acute lymphoblastic leukemia when their immune system was overstimulated [10]. However, we could not assess leukemia transformation through serial transplantation because of the rarity of this phenotype in the Mll-AF4+VEC-Cre+ cohort (Figure 3A). Overall, these results suggest that maternal immune activation through a single dose of poly(I:C) is not sufficient to trigger MLL-AF4-driven leukemogenesis.

### Mll-AF4+VEC-Cre+ aging mice have a higher proportion of CD3+ T-lymphoid cells in the spleen

**Figure 4:**
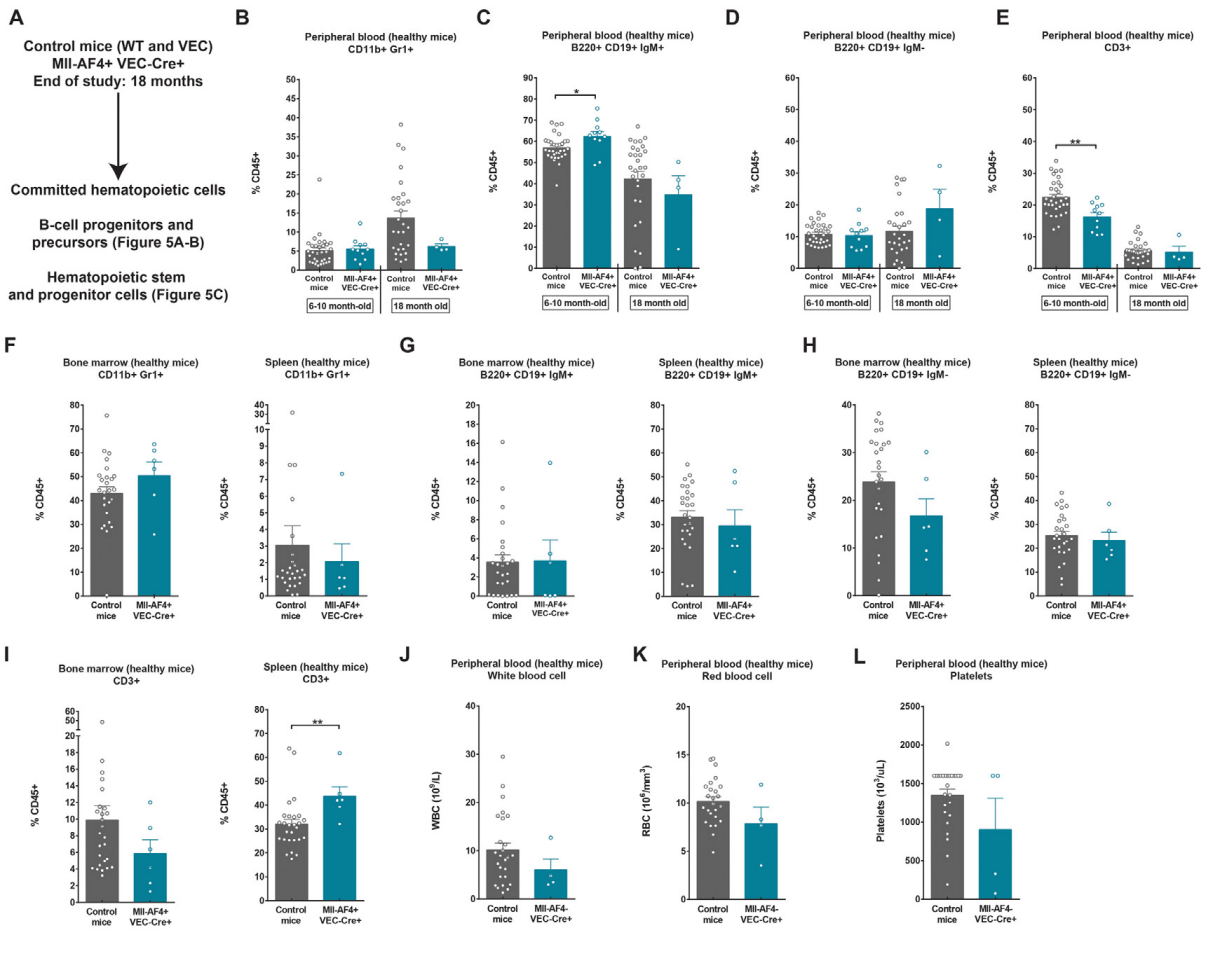
Mll-AF4+VEC-Cre+ aging mice have a higher proportion of CD3+ T-lymphoid cells in the spleen. **(A)** Experimental layout. Proportions of **(B)** CD11b+Gr1+, **(C)** B220+CD19+IgM+, **(D)** B220+CD19+IgM–, and **(E)** CD3+ cells in the peripheral blood of the control and Mll-AF4+VEC-Cre+ aged cohort at two time points. Proportions of **(F)** CD11b+Gr1+, **(G)** B220+CD19+IgM+, (**H)** B220+CD19+IgM–, and **(I)** CD3+ in the bone marrow and spleen of the control and Mll-AF4+VEC-Cre+ cohort at the end of study (18 months old). Peripheral blood counts of **(J)** white blood cells, **(K)** red blood cells, and **(L)** platelets of the control and Mll-AF4+VEC-Cre+ cohort at the end of study (18 months). Statistical differences were assessed using a nonparametric Mann–Whitney *U* test with a bilateral *p* value (*p < 0.05, **p < 0.01, ***p < 0.001).

Our previous studies have focused mainly on the changes conferred by Mll-AF4 in embryos and younger mice, but we never assessed the effect of Mll-AF4 on the hematopoietic compartment in Mll-AF4+VEC-Cre+ aging mice. We monitored their blood composition throughout adult life (6–10 and 18 months) and sacrificed the Mll-AF4+VEC-Cre+ and control cohorts at 18 months of age (Figure 4A). The proportion of CD11b+Gr1+ myeloid cells in the peripheral blood did not change with age (Figure 4B). We noted an increase in B220+CD19+IgM+ mature B-lymphoid cells in 6- to 10-month-old mice, which normalized upon aging (Figure 4C). This B-lymphoid bias is in line with our previous studies 4, 7. No significant difference was observed for B220+CD19+IgM– immature B-lymphoid cells in the peripheral blood, regardless of Mll-AF4 expression (Figure 4D). There was, however, a small decrease in CD3+ T-lymphoid cells in the 6- to 10-month-old mice (Figure 4E). At 18 months, we also analyzed the bone marrow and spleen. There was no significant difference in the proportion of myeloid cells and B-lymphoid cells in the bone marrow and spleen of aging Mll-AF4+VEC-Cre+ compared with control mice (Figure 4F–H). Interestingly, aging Mll-AF4+VEC-Cre+ mice showed an increase in CD3+ T-lymphoid cells in the spleen (Figure 4I). The peripheral blood counts (white blood cell, red blood cell, and platelet) were similar between 18-month-old control and Mll-AF4+VEC-Cre+ mice (Figure 4J–L). Overall, these results support the increased B-lymphoid potential conferred by Mll-AF4 in younger mice, which becomes undetectable in aging mice. Instead, aging mice have a higher proportion of CD3+ T-lymphoid cells, which may contribute to a hematological malignancy in a small subset [4].

### Mll-AF4 maintains the progenitor B-cell compartment in the spleen of aging mice and decreases the bone marrow HSC-MPP pool

**Figure 5:**
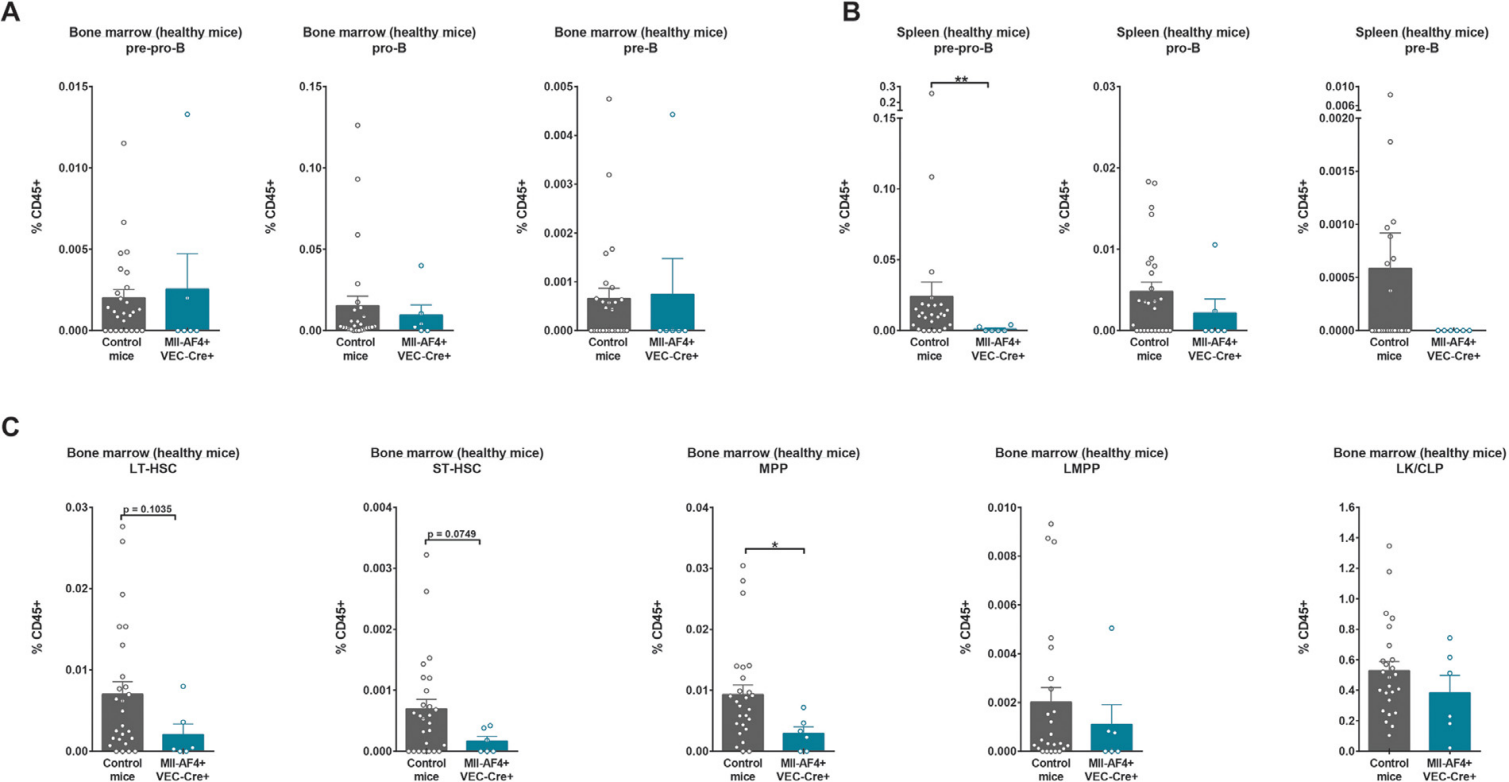
Mll-AF4 maintains the progenitor B-cell compartment in the spleen of aging mice and decreases the bone marrow HSC-MPP pool. Proportions of pre-pro-B cells (B220+CD19–ckit+ IL7R+CD43+CD24–IgM–); pro-B cells (B220+CD19+ckit+IL7R+CD43+CD24+IgM–); and pre-B cells (B220+CD19+ckit+IL7R+CD43–CD24+IgM–) in the **(A)** bone marrow and **(B)** spleen of the control and Mll-AF4+VEC-Cre+ cohort at the end of study (18 months). **(C)** Proportions of LT-HSC (LSK CD34–FLT3–IL7R–CD48–CD150+); ST-HSCs (LSK CD34+FLT3–IL7R–CD48–CD150+); MPPs (LSK CD34+FLT3–IL7R–CD48+), LMPPs (LSK FLT3+) and LK-CLPs (Lineage–ckit+/low Sca1–/low) in the bone marrow of the control and Mll-AF4+VEC-Cre+ cohort at the end of study (18 months). Statistical differences were assessed using a nonparametric Mann–Whitney *U* test with a bilateral *p* value (*p < 0.05, **p < 0.01, ***p < 0.001).

We also verified the B-cell progenitor and precursor populations in the bone marrow and spleen of aging mice: pre-pro-B, pro-B, and pre-B (Figure 5A,B). The proportions of pre-pro-B, pro-B, and pre-B lymphoid cells were similar in the bone marrow of Mll-AF4+VEC-Cre+ and control aging mice (Figure 5A). Interestingly, we did not detect many pre-pro-B and pre-B lymphoid cells in the spleen, whereas pro-B lymphoid cells were maintained (Figure 5B). These results suggest that Mll-AF4 favors the maintenance of pro-B cells in the spleen compared with pre-pro-B and pre-B cells, but this is not sufficient to drive a full-blown leukemia. In the bone marrow hematopoietic stem and progenitor cell compartment, mice that expressed Mll-AF4 had a trend toward fewer long-term HSCs and short-term HSCs and significantly fewer MPPs (Figure 5C). The proportions of LMPPs and LK-CLPs were similar for Mll-AF4+VEC-Cre+ and control aging mice (Figure 5C). These results suggest that Mll-AF4 can maintain the more mature progenitor populations with age, but has a potentially negative effect on the maintenance of the more primitive HSC-MPP compartment.

## Discussion

There is now abundant epidemiological and functional evidence suggesting that an overstimulation of the immune system can accelerate leukemia development in the pediatric population [9], which is being validated in multiple mouse models of acute lymphoblastic leukemia 10, 11. Given the early onset and high concordance of infant t(4;11) MLL-AF4 acute leukemia in monozygotic twin studies, it is expected that most of the events leading to leukemogenesis will occur during fetal life [1]. The aim of our study was therefore to determine whether exposure of fetal cells to inflammatory signals can also contribute to the development of MLL-AF4-driven infant leukemia. We wanted to assess first if the target cell compartment (FL Mll-AF4+ LSK cells) can directly respond to a viral or bacterial mimic and, second, if maternal immune activation can lead to full-blown t(4;11) MLL-AF4 acute leukemia.

We exposed FL Mll-AF4+ LSK cells directly to two different stimuli: the viral mimic poly(I:C) and the bacterial mimic LPS. FL Mll-AF4+ LSK cells express both *Tlr3* and *Tlr4*, the receptors recognized by poly(I:C) and LPS, respectively. An exposure to poly(I:C) and LPS mimics did not affect the proliferation, apoptosis, or clonogenicity of FL Mll-AF4+ LSK cells, nor did it induce a differentiation bias; however, LPS increased the proliferation within colonies derived from FL Mll-AF4+ LSK cells placed into B-lymphoid conditions, whereas poly(I:C) increased proliferation in both myeloid and B-lymphoid conditions. This suggests that viral and bacterial mimics target proliferation at the more mature end of the hematopoietic spectrum. Interestingly, LPS exposure upregulated the expression of *Tlr3* which is recognized by poly(I:C), and an exposure to poly(I:C) increased the expression of *Tlr9, Mda5,* and *Rig1*. These results indicate that FL Mll-AF4+ LSK cells are able to activate the Toll-like receptor signaling pathway upon stimulation with poly(I:C) or LPS.

T(4;11) MLL-AF4 acute leukemia has a very unique gene expression signature, which includes important players such as *Cdk6, Bcl2, Hoxa9,* and *Meis1* 19, 20. We assessed the expression of 16 known candidate genes in FL Mll-AF4+ LSK cells exposed to poly(I:C) or LPS: *Mll-AF4, Mll, Af4, Bcl2, Cdk6, E2a, Flt3, Hmga2, Hoxa9, Ikaros, Il7r, Lmo2, Mcl1, Meis1, Pax5* and *Twist1*. FL Mll-AF4+ LSK cells exposed to poly(I:C) upregulated *Mll-AF4, Mll, Af4, Cdk6, Hmga2, Hoxa9, Il7r, Lmo2,* and *Mcl1* and lost *Flt3* expression. LPS, on the other hand, had little effect, apart from a slight downregulation of *Bcl2* and *Meis1*. The lack of effect of LPS on the MLL-AF4 gene expression signature could be due to the lower expression of its receptor, *Tlr4*, compared with the poly(I:C) receptor *Tlr3*. Importantly, the upregulation of the MLL-AF4 signature in response to poly(I:C) was dependent on Mll-AF4 expression as control cells did not show this effect. Our results suggest that FL Mll-AF4+ LSK cells are potentially more sensitive to a viral mimic, and this can lead to an overexpression of a subset of MLL-AF4 signature genes.

Our results clearly illustrate the ability of HSPCs to respond directly to poly(I:C) in vitro; however, it is known that poly(I:C) can induce a more systemic interferon response, the complexity of which cannot be modeled in vitro. We therefore proceeded to stimulating the pregnant dam with poly(I:C) to mimic an in vivo inflammatory response to a viral stimulus. It is currently unknown if poly(I:C) can penetrate the placental barrier, suggesting that FL Mll-AF4+ LSK cells are potentially protected from poly(I:C). However, a maternal immune activation can compromise the placental barrier and allow passage of maternally derived cytokines to the fetus, which can in turn activate its immune response (reviewed in [21]). The plasma of 10-day-old mouse pups exposed to poly(I:C) during gestation was shown to have increased levels of certain cytokines (e.g., IL-2, IL-5, and IL-6), suggesting that the inflammatory effect mediated by the exposure of pregnant dams to poly(I:C) can persist after birth [22]. Cytokine expression in fetal tissue after a maternal immune activation is also very dynamic: some changes occur quickly (6 hours), whereas some changes become noticeable after a longer period (24 hours) [23]. Given the impact of a viral stimulation on brain development [24], we injected pregnant dams with a single dose of poly(I:C) to avoid complications in the offspring. Maternal immune activation did not lead to the development of a t(4;11) MLL-AF4 acute leukemia in the offspring. Two Mll-AF4+VEC-Cre+ mice died at an old age (564 and 516 days) and presented with a myelodysplastic syndrome. One mouse was found dead 10 days after birth and presented with an expansion of B220+CD19−IgM− cells. However, we could not assess the leukemia transformation given the low amount of material available, so we cannot conclude that the cause of death is a hematological malignancy. These results are in line with other studies indicating that animal models that rely solely on Mll-AF4 and mouse cells do not develop a full-blown leukemia 2, 4. MLL-AF4-driven leukemogenesis in mice appears to require additional factors to complete the transformation process. These factors could be cell intrinsic through the aberrant expression of a particular gene such as the reciprocal fusion AF4-MLL, but could also come from the microenvironment 25, 26, 27.

Given the negative results of the adult cohort, we did not investigate the types of cytokines released upon the maternal immune activation. Instead, we analyzed the hematopoietic composition in aging mice to assess hematopoietic differentiation and composition of the progenitor and precursor B-lymphoid and hematopoietic stem and progenitor pools. We found an increased proportion of B220+CD19+IgM+ B-lymphoid cells and a decrease in CD3+ T-lymphoid cells in the peripheral blood of younger Mll-AF4+VEC-Cre+ mice compared with control mice. However, this normalized with age, supporting the notion that there is a developmental window of opportunity in which the disease can arise [4]. Aging Mll-AF4+VEC-Cre+ mice displayed a higher proportion of CD3+ T-lymphoid cells in their spleen instead. The bone marrow progenitor and precursor B-lymphoid pools were similar in control and Mll-AF4+VEC-Cre+ mice, but pro-B cells were preferentially maintained in the spleen. This suggests that Mll-AF4 confers an advantage in maintaining the pro-B-lymphoid cell pool, which is similar to the leukemic blasts observed in patients. However, there is a missing component that could drive this to a full-blown leukemia. The HSC-MPP frequency was reduced in Mll-AF4+VEC-Cre+ aging mice compared with control mice, whereas the LMPPs and LK-CLPs remained similar. This suggests that Mll-AF4 can maintain the hematopoietic progenitor pool better than the stem cell pool during aging. The cohort of aged mice had initially received a single dose of poly(I:C) via the pregnant dam; however, because of the mild effect of poly(I:C) initially, it is unlikely that the aging symptoms are influenced by poly(I:C), but instead are entirely due to the influence of Mll-AF4.

Overall, this study shows that FL Mll-AF4+ LSK cells are sensitive to viral and bacterial mimics, but the MLL-AF4 gene expression signature only changes with the viral mimic. The maternal immune activation with a single exposure to poly(I:C) is not enough to trigger MLL-AF4 leukemogenesis. Future studies will be necessary to assess if a constant maternal immune activation of the pregnant dam and offspring can trigger MLL-AF4-driven leukemogenesis and, if so, what pathogens are involved.

## Appendix Supplementary materials

Image, application 1

## Footnotes

Supplementary material associated with this article can be found, in the online version, at https://doi.org/10.1016/j.exphem.2019.07.004.
